# Challenging intrahepatic laparoscopic cholecystectomy: a case report

**DOI:** 10.1093/jscr/rjaf667

**Published:** 2025-08-29

**Authors:** Nouf Tuhaif Algahtani, Ahmad Saad Alshehri, Areej A Alhumaid, Bandar Nasser Alharthi

**Affiliations:** Department of Surgery, King Abdulaziz Medical City, Ministry of National Guard Health Affairs, Ar Rimayah District, PO Box 22490, Riyadh 11426, Riyadh Province, Saudi Arabia; College of Medicine, King Saud bin Abdulaziz University for Health Sciences (KSAU-HS), Sheikh Jaber Al-Sabah Road, Khashm Al An District, PO Box 3660, Riyadh 11481, Riyadh Province, Saudi Arabia; Department of Surgery, Breast Surgical Oncology, King Abdullah Bin Abdulaziz University Hospital Princess Norah university, Airport Road, King Khalid International Airport, Riyadh 11564, Riyadh province, Saudi Arabia; Department of Surgery, Breast, Endocrine and Laparoscopic Surgery, King Abdullah Bin Abdulaziz University Hospital Princess Norah university, Airport Road, King Khalid International Airport, Riyadh 11564, Riyadh Province, Saudi Arabia

**Keywords:** intrahepatic, gallbladder, cholelithiasis, cholecystitis, laparoscopy

## Abstract

There have been reports of gallbladders in several ectopic locations. A gallbladder that is intrahepatic is one that is located along the anterior inferior right lobe of the liver or within the liver parenchyma. A 39-year-old male patient presented with symptoms that led to a gallbladder origin and was worked up using ultrasonography, which confirmed the presence of a contracted gallbladder with multiple stones. The patient was booked electively for a laparoscopic cholecystectomy. Diagnostic laparoscopy was utilized and revealed only the lower end of the gallbladder, while the rest was embedded within the liver parenchyma. After resection, the gallbladder was sent to pathology and came back as chronic cholecystitis. The patient was discharged with an unremarkable postoperative period. Intrahepatic gallbladders can be very challenging to locate during a laparoscopic procedure, but can still be managed without converting to an open technique.

## Introduction

Intrahepatic ectopic gallbladders are one of the surgical challenges encountered during a laparoscopic cholecystectomy. An intrahepatic gallbladder often exhibits impaired function, which may lead to stasis and gallstone formation [1, 2]. Thus, patients with intrahepatic gallbladders are at higher risk than others to develop complications of cholelithiasis, such as cholecystitis [3]. Herein, we describe a successful surgical treatment of a rare case of chronic cholecystitis in a patient with a complete fundal intrahepatic gallbladder.

## Case presentation

A 39-year-old male patient presented with symptomatic gallstones, for which an ultrasound sonograph confirmed the presence of contracted gallbladder with multiple stones. The largest measuring 2.1 cm, with preserved gallbladder thickness, no pericholecystic collection, and no previous admissions to the emergency department.

**Figure 1:**
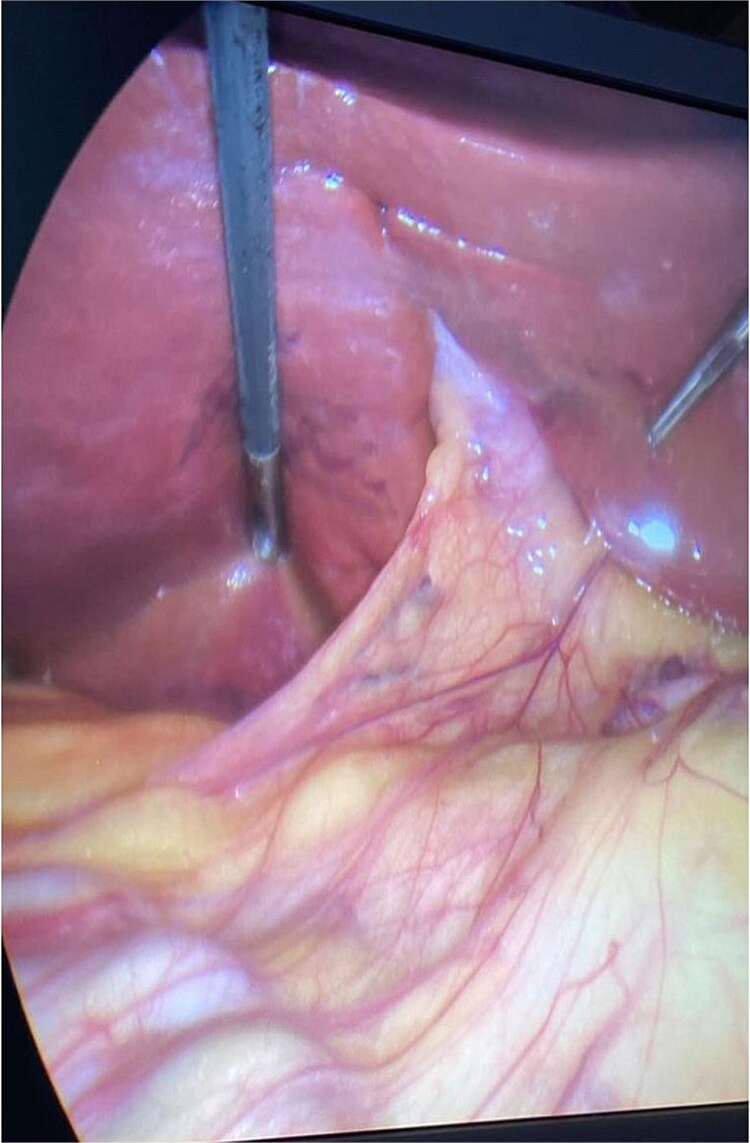
Intra-operative view showing the embedded gallbladder while utilizing diagnostic laparoscopy.

**Figure 2:**
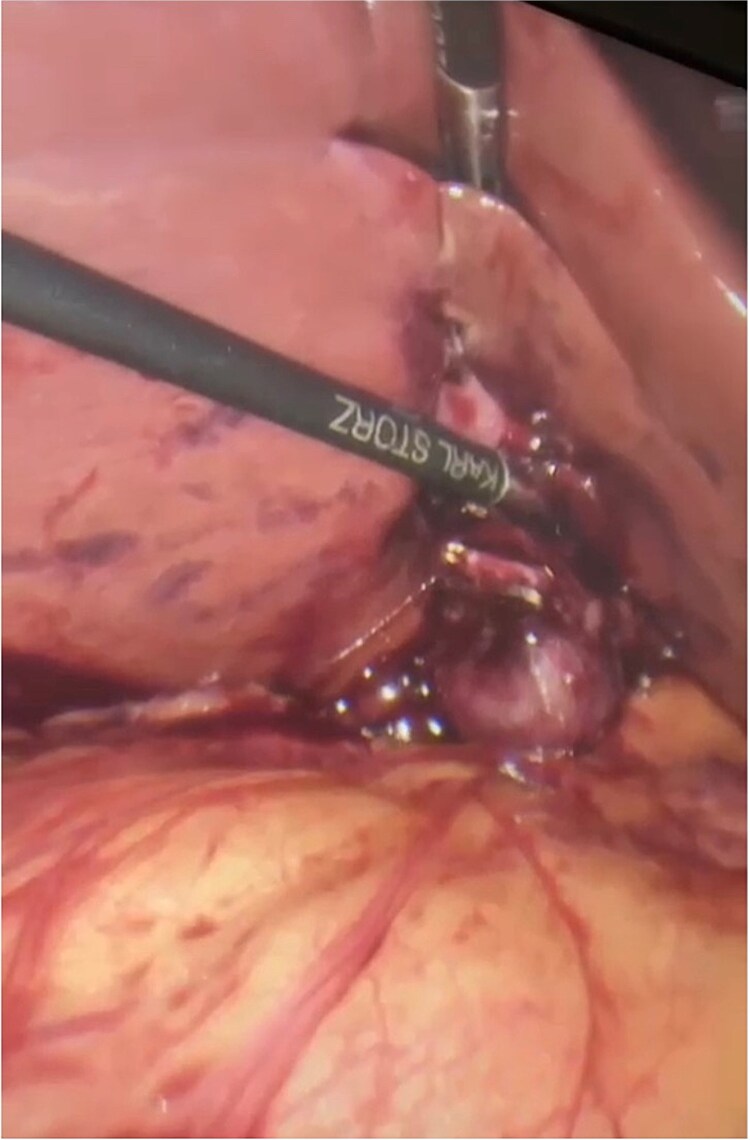
Intraoperative view showing the embedded gallbladder during dissection.

The patient booked electively for a laparoscopic cholecystectomy. Pneumoperitoneum was established by open technique, and four ports were inserted during the entrance to the abdomen. Furthermore, only the lower end of the gallbladder could be visualized, while the rest of gallbladder was embedded within the liver parenchyma. This can be appreciated in Figs 1 and  2. Critical view of safety was achieved, and the cystic duct and cystic artery were clipped. The fundus of the gallbladder was appreciated embedded within the liver, and after complete dissection over the superior fissure, the fundus was exposed. The anterior liver parenchyma and liver bridge were dissected to expose the fundus, and then dissection proceeded. One drain was inserted and removed after 2 days. The patient was discharged with an unremarkable postoperative period. The pathology specimen came back as chronic cholecystitis with no dysplasia or malignancy, containing multiple stones.

## Discussion

Generally, the gallbladder is situated on the plane of the interlobar fissure, beneath the right lobe of the liver [4]. Embryologically, the gallbladder forms during the organogenesis of the liver, biliary system, and ventral pancreas around the fourth week of pregnancy from the caudal bud, which itself develops from the hepatic diverticulum of the primitive midgut [5]. The gallbladder can grow ectopically in multiple different locations, with the most commonly seen ectopic gallbladder locations being: intrahepatic, transverse, retrohepatic, and under the left lobe of the liver [4]. It has been estimated that 0.1%–0.7% of gallbladder cases are ectopic [6]. And of the aforementioned percentage, intrahepatic gallbladders are the second most common site in which ectopic gallbladders are found [7]. Herein, we presented a successful surgical treatment of a rare case of chronic cholecystitis in a patient with a complete fundal intrahepatic gallbladder.

Cholelithiasis is a general term that can refer to any condition brought on by gallstones. Patients with gallstone disease frequently exhibit biliary colic symptoms, such as intermittent attacks of persistent, sharp, abdominal pain in the right upper quadrant (RUQ), which is frequently accompanied by nausea and vomiting, as well as normal physical examination findings and laboratory test results. Diaphoresis, nausea, and vomiting are symptoms that may also develop [8]. The most effective imaging modality for the diagnosis of cholelithiasis is ultrasonography [9]. And the intervention of choice is a laparoscopic cholecystectomy, which is regarded as the gold standard of treatment for its minimal invasiveness and lower hospital length of stay [10]. The high prevalence of cholelithiasis in the intrahepatic gallbladder increases the likelihood of symptomatic patients and can impair imaging diagnosis [11]. Nevertheless, ultrasonography confirmed the presence of gallbladder stones in our case, with the largest measuring 2.1 cm, with preserved gallbladder thickness. Informed consent was obtained in clinic for a laparoscopic possible open cholecystectomy, during surgery, the infundibulum was identified, and the intrahepatic gallbladder distinction was made.

Despite its rarity, it is crucial for any surgeon operating laparoscopically on gallbladder to be aware of its unusual ectopic positions. Due to biliary stasis, ectopic gallbladders are at a higher likelihood to co-exist with cholelithiasis, making them a torsion risk if suspended on mesentery, and even having the potential to herniate through the foramen of Winslow [3]. The majority of these instances will therefore proceed to surgery. This may result in more challenges during surgery, especially if the likelihood of an ectopic gallbladder is not known. Although case reports have emphasized the value of diagnosing ectopic gallbladders prior to surgery, unfortunately, this was not the case in our situation, since most of our cases are operated on based on a clinical diagnosis [7,12]. Despite the fact that laparoscopic cholecystectomy surgeries for ectopic gallbladders seem to be increasingly common, our experience shows that open surgery employing a straightforward drainage of the gallbladder with the electrocautery method can be carried out with excellent results even if the gallbladder’s position is determined intra-operatively. Furthermore, there are no approved reports discussing the safety of laparoscopic procedures in challenging intrahepatic gallbladders to date, so open surgery should still be considered, especially if the anatomy is not clear [13].

## Conclusion

The intrahepatic gallbladder is a rare condition that can be encountered during a laparoscopic cholecystectomy. The pre-operative identification and detection of the ectopic gallbladder can be challenging, and experience in dealing with such an unusual finding is a key factor in determining outcomes.
